# Factors increasing the risk for food addiction in Ecuadorian students

**DOI:** 10.3389/fpsyt.2023.1214266

**Published:** 2024-01-03

**Authors:** Geovanny Genaro Reivan Ortiz, Ximena Campoverde, Juan Vinañzaca, Johanna Estrada, Rafael Yanza, Roser Granero

**Affiliations:** ^1^Laboratory of Basic Psychology, Behavioral Analysis, and Programmatic Development (PAD-LAB), Catholic University of Cuenca, Cuenca, Ecuador; ^2^Catholic University of Cuenca, Cuenca, Azuay, Ecuador; ^3^Autonomous University of Barcelona, Barcelona, Spain

**Keywords:** food addiction, eating disorder, path analysis, emotion regulation, Ecuadorians

## Abstract

**Background:**

Food addiction (FA) is a construct that has gained interest in recent years, but its relevance in the Ecuadorian population has not yet been explored. The aims of this study were to explore the differences in the psychological profile (including FA) between university students from Ecuador and to identify the underlying structure of the relationships of the FA severity level through a mediational model.

**Methods:**

The sample consisted of 972 university students, women and men (mean age: 20.1 years old, SD = 2.6), recruited from four Ecuadorian regions. The assessment tools included a unidimensional scale of FA, eating-related measures, emotion regulation state, impulsivity, and psychopathology state. Path analysis modeled the direct and indirect effects explaining the FA severity level.

**Results:**

The results indicated that higher psychopathological levels were associated with FA. Similarly, no differences in FA were observed between the Ecuadorian regions. The path analysis suggested that older age, female sex, and higher difficulties in emotional regulation, impulsivity, negative mood, and anxiety trigger disordered eating; subsequently, more impaired eating behavior impacted the FA level.

**Conclusion:**

FA is a complex clinical entity that includes multiple components related to eating disorders (EDs) and other mental health problems. The results of this study provide empirical knowledge for designing evidence-based prevention and treatment strategies.

## Introduction

Obesity has become a priority condition in the world due to its high prevalence and various chronic diseases associated with it (1, 2). Additionally, obesity is associated with a poorer quality of life and leads to high public health costs (3).

In Ecuador, 6 out of 10 people have presented problems of overweight (BMI > 25) and obesity (BMI > 30), being more prevalent during adolescence and early adulthood. Women (65.5%) have the highest frequency than men (34.5%). Despite the country's security measures, the number of people with overweight/obesity is increasing (4). This topic shows the need to go even deeper into the study of the various factors that may be involved in its development in order to improve prevention and treatment programs.

As indicated, studies show that obesity is a problem in the adult population, especially women, with increasing frequency in older age groups (5). Similarly, empirical evidence mentions that this higher trend in women than in men is positively correlated with age, and there may be a greater vulnerability to suffering from disordered eating behaviors (6–9).

Food addiction (FA) has become a widely recognized issue as one of the key players that can explain the processes or behaviors that contribute to the development and maintenance of obesity and eating disorders (EDs) (10–15). Recently, high levels of FA have been found to be the most important psychological factor in unsuccessful weight loss (16). Despite its importance, FA is poorly studied in the Ecuadorian population.

FA is defined as hedonic eating behavior involving the consumption of highly palatable foods (i.e., foods high in salt, fat, and sugar) in quantities beyond homeostatic energy requirements (17). The FA model has been proposed considering the similarities between the processes found in substance abuse disorders (18) and the consumption of certain foods that have been described as potentially addictive, such as sweet, salty, fatty, and processed foods (17, 19, 20). Similar to the mechanism described in addictive behaviors, FA is associated with the compulsive search for and consumption of these foods despite their negative consequences, the presence of tolerance, withdrawal, and activation in the same brain areas. The effects involve activity in the *nucleus accumbens*, including activation of the brain's dopamine and opioid signaling system (21).

Several variables have been closely linked to FA, emerging as risk patterns. A risk pattern refers to any identifiable trait or situation in an individual or group that is known to increase the likelihood of experiencing, developing, or being particularly vulnerable to a specific pathology or disease; for example, impulsivity (22–26), denoted as a personality trait characterized by fast, unexpected and excessive reactions. Studies indicate that impulsivity increases the risk of FA (27, 28).

Moreover, studies indicate that FA presents significantly higher scores when associated with anxiety (29–33). For some people, compulsive eating is a way of coping with anxiety (30, 34). Similarly, a large body of literature indicates that anxiety and other variables and affective dysregulation (35–41) have a mediating effect on many mental disorders, such as EDs.

Another indirect and etiopathogenic factor of FA is emotional dysregulation, which refers to the difficulty in the way people experience and express their emotions (42). Precisely, high rates of emotional dysregulation have been found in FA (18, 43, 44). Although the precise role of this construct needs to be further explored, FA behaviors could also be used to cope with negative affects, such as fear, sadness, anger, disgust, and guilt (45–47).

To summarize, higher levels of the aforementioned variables may be associated with addiction to food and, hence, with elevated eating psychopathology, greater preponderance of pathognomonic features associated with EDs, and higher BMI (48, 49).

As previously stated, FA has been minimally researched in Ecuador, although research on the effects of hypercaloric diets has gained interest in Latin America (50, 51). It has been found that, in the young population, it is highly influenced by the eating context and FA (49, 52, 53). However, there are no studies that examine the prevalence of FA among Ecuadorian young adults, as well as studies that explore the influence of various factors and interactions on its development.

Despite the efforts of health organizations imposed by the government in the incorporation of preventive strategies for obesity, the high rates and comorbidities associated with it (high blood pressure, high cholesterol, type 2 diabetes, coronary diseases, attacks or stroke, gallbladder diseases, etc.), mortality rates continue to rise in the Ecuadorian population. This behavior constitutes an alarm signal for the region.

This reality is not unfamiliar to the university sector. Research reveals that Ecuadorian students are overweight and obese, sedentary for extended periods, engage in limited physical activity, consume alcohol, get inadequate sleep, lack discipline in their eating habits, and consume ultra-processed foods that are low in nutritional value but high in salt, saturated fats, sugar, and even exhibit FA tendencies. This situation is the impetus for our research, which seeks to ascertain the prevalence of FA in young Ecuadorian adults and explore the various contributing factors and their interplay in its onset.

Therefore, this study proposed two objectives: to assess the psychological characteristics associated with the different results of the AF screening (whether absent, probable, or present) and to formulate a predictive model that can measure the severity of FA in the Ecuadorian population.

We propose that the screening for addiction to present food will have a worse psychological profile in Ecuadorians. Finally, despite the fact that the literature indicates an association between the etiological factors in isolation, the total composition of these has not been investigated through an integrated model; for this, we propose the hypotheses according to structural models proposed according to the study of Munguía et al. (54) and Wolz et al. (55), in this way, we organize the routes, of their risk patterns: elevated levels of FA are associated with increased: impulsivity, anxiety, and difficulties in emotional regulating; older age is linked to increased impulsivity and emotional imbalance; and the female sex is often associated with more challenges in eating behaviors.

## Materials and methods

### Participants

The sample was selected using a non-probabilistic convenience design. Based on the researchers' access to potential participants, 2,654 students from mid-level undergraduate programs, such as Business Administration, Accounting, Psychology, Nursing, and Medicine, were invited. These students were from various departments and extensions of the Catholic University throughout Ecuador. The constituted cities, whose participants are native, were made up of the following regions: Galapagos (Isabela Island), Coast (Machala, Pasaje, and Guayaquil), Mountain (Quito and Cuenca), and East (Macas, Sucua, Limón Indaza, and Gualaquiza). Participants ranged in age from 17 to 29 and belonged to the middle socioeconomic class. Only those who agreed to participate underwent evaluation. The ultimate sample included 972 participants, comprising 688 women and 284 men, representing all four regions of Ecuador: Galapagos, Coast, Mountain, and East. Detailed breakdowns are provided in Table 1. The participants were recruited as volunteers, and all of them signed the informed consent. Participants under 18 years of age who participated in the study also presented informed consent signed by their parents. No compensation was awarded for participating in the study. Assessed by a questionnaire adapted from the DSM-5 Structured Clinical Interview for Eating Disorders (SCID-5), no participant reported having had an eating disorder (ED) in their lifetime. Participants were not evaluated for any other medical or psychiatric problem.

**Table 1:** Descriptive for the variables of the study.

	**Total (*n =*** **972)**		**Galapagos (*n =*** **11)**		**Coast (*n =*** **291)**		**Mountain (*n =*** **596)**		**East (*n =*** **74)**			
	***n***	***%***	***n***	***%***	***n***	***%***	***n***	***%***	***n***	***%***	**χ^2^**	***p***
**Sex**												
Female	688	70.8%	8	72.7%	220	75.6%	405	68.0%	55	74.3%	6.04	0.110
Male	284	29.2%	3	27.3%	71	24.4%	191	32.0%	19	25.7%		
**Marital**												
Single	906	93.2%	9	81.8%	275	94.5%	552	92.6%	70	94.6%	4.18	0.653
Married	62	6.4%	2	18.2%	15	5.2%	41	6.9%	4	5.4%		
Divorced	4	0.4%	0	0.0%	1	0.3%	3	0.5%	0	0.0%		
***Mean***	***SD***	***Mean***	***SD***	***Mean***	***SD***	***Mean***	***SD***	***Mean***	***SD***	***F-stat***	***p***
Age (years-old)	20.13	2.63	20.27	4.36	20.09	2.58	20.14	2.60	20.23	2.76	0.07	0.975
EDI-3 total	100.31	38.24	83.09	49.31	99.97	39.05	100.89	37.50	99.59	39.40	0.81	0.491
DEBQ total	1.77	1.38	1.91	1.45	1.77	1.33	1.74	1.39	1.97	1.42	0.66	0.578
UPPS-P total	25.04	8.24	21.91	10.63	26.25	7.92	24.75	8.26	23.09	8.40	4.28	**0.005** ^*^
DERS total	17.66	6.37	16.00	7.87	18.36	6.06	17.48	6.42	16.64	6.76	2.22	0.084
PSS total	12.99	2.39	12.73	3.47	13.20	2.16	12.91	2.42	12.80	2.74	1.18	0.317
GADS total	26.04	4.93	24.36	6.74	26.78	4.49	25.80	4.96	25.38	5.70	3.57	0.014^*^
PANAS total	8.84	3.25	7.82	3.74	9.13	3.16	8.76	3.26	8.49	3.39	1.54	0.202
YFAS total	52.27	18.60	50.00	24.27	53.78	18.21	51.66	18.65	51.59	18.85	0.94	0.422

SD, standard deviation. ^*^Significant comparison. EDI, Eating Disorders Inventory; DEBQ, Dutch Eating Behavior Questionnaire; UPPS-P, Impulsive Behavior Scale; DERS, Difficulties in Emotional Regulation Scale; PSS, Perceived Stress Scale; GADS, Goldberg Anxiety and Depression Scale; PANAS, Positive and Negative Affect Scale; YFAS, Yale Food Addiction Scale.

### Assessment

In addition to a targeted sociodemographic questionnaire capturing key variables like sex, marital status, and age, other instruments were also employed. A detailed description of all study measures can be found in Table 1, while the correlation matrix for these variables is presented in Table 2.

**Table 2:** Comparison between the FA screening groups.

	**FA absent (*n =*** **723)**		**FA probable (*n =*** **172)**		**FA present (*n =*** **77)**		**FA absent vs. FA probable**		**FA absent vs. FA present**		**FA present vs. FA probable**	
	***n***	***%***	***n***	***%***	***n***	***%***	***p***	***|C-V|***	***p***	***|C-V|***	***p***	***|C-V|***
**Sex**												
Female	514	71.1%	126	73.3%	48	62.3%	0.572	0.019	0.110	0.056	0.083	0.110
Male	209	28.9%	46	26.7%	29	37.7%						
**Marital**												
Single	671	92.8%	159	92.4%	76	98.7%	0.570	0.035	0.141	0.070	0.052	0.126
Married	48	6.6%	13	7.6%	1	1.3%						
Divorced	4	0.6%	0	0.0%	0	0.0%						
**Origin**												
Galapagos	9	1.2%	1	0.6%	1	1.3%	0.197	0.072	0.995	0.009	0.503	0.097
Coast	207	28.6%	63	36.6%	21	27.3%						
Mountain	450	62.2%	97	56.4%	49	63.6%						
East	57	7.9%	11	6.4%	6	7.8%						
***Mean***	***SD***	***Mean***	***SD***	***Mean***	***SD***	***p***	***|d|***	***p***	***|d|***	***p***	***|d|***
Age (years-old)	20.11	2.57	20.23	2.91	20.16	2.52	0.577	0.05	0.879	0.02	0.832	0.03
EDI-3 total	85.62	27.36	133.3	27.85	164.6	33.56	**<0.001** ^*^	**1.73** ^†^	**<0.001** ^*^	**2.58** ^†^	**<0.001** ^*^	**1.02** ^†^
DEBQ total	1.65	1.34	1.95	1.36	2.45	1.54	**0.009** ^*^	0.22	**<0.001** ^*^	**0.56** ^†^	**0.007** ^*^	0.34
UPPS-P total	24.60	8.39	25.83	6.98	27.47	9.00	0.076	0.16	**0.004** ^*^	0.33	0.146	0.20
DERS total	17.33	6.54	18.63	5.38	18.57	6.59	**0.016** ^*^	0.22	0.104	0.19	0.943	0.01
PSS total	12.71	2.46	13.45	1.72	14.57	2.15	**<0.001** ^*^	0.35	**<0.001** ^*^	**0.81** ^†^	**<0.001** ^*^	**0.57** ^†^
GADS total	25.48	5.08	27.01	3.72	29.16	4.31	**<0.001** ^*^	0.34	**<0.001** ^*^	**0.78** ^†^	**0.001** ^*^	**0.53** ^†^
PANAS total	8.67	3.31	9.35	2.82	9.34	3.45	**0.013** ^*^	0.22	0.085	0.20	0.980	0.00

SD, standard deviation; FA, food addiction; EDI, Eating Disorders Inventory; DEBQ, Dutch Eating Behavior Questionnaire; UPPS-P, Impulsive Behavior Scale; DERS, Difficulties in Emotional Regulation Scale; PSS, Perceived Stress Scale; GADS, Goldberg Anxiety and Depression Scale; PANAS, Positive and Negative Affect Scale; YFAS, Yale Food Addiction Scale. ^*^Bold, Significant comparison.^†^Bold, effect size within the ranges mild-moderate to large-high.

*Yale Food Addiction Scale 2.0 YFAS 2.0* (56) is a 35-item self-report questionnaire to measure addictive eating behaviors during the previous 12 months. The original instrument (YFAS) was based on the substance dependence criteria of the Diagnostic and Statistical Manual of Mental Disorders (DSM-IV-TR) (57) and was adapted to the context of food consumption. The YFAS 2.0 is based on the DSM-5 (58) and assesses 11 symptoms. The score produces two measures: (a) a continuous symptom count score that reflects the number of diagnostic criteria met (ranging from 0 to 11) and (b) an FA threshold based on the number of symptoms (at least 2) and self-reported clinically significant impairment or distress. This final measure allows for binary classification of FA (present vs. absent). In addition, according to the revised taxonomy of the DSM-5 (58), it is possible to establish cut-off points for severity: mild (2–3 symptoms), moderate (4–5 symptoms), and severe (6–11 symptoms). The FA risk groups were calculated according to a previous study (54) as follows: absent FA, those who do not have any diagnostic criteria in the YFAS 2.0; probable FA, those who meet one diagnostic criterion and those who have two or more criteria but do not present clinical deterioration; and present FA, those who have two diagnostic criteria and also present clinical deterioration. Thus, the probable group of FA will correspond to the clinical concept of high or subthreshold risk, that is, patients who do not strictly meet the diagnostic criteria of taxonomy but who present symptoms. Subthreshold psychiatric symptoms do not meet the full criteria for a particular disorder in a reference diagnostic taxonomy (such as Axis I disorders within the DSM) but do present with significant clinical deterioration. In some cases, subthreshold symptoms are more common than their respective Axis I disorders, and empirical research has suggested that these groups are associated with greater disability and many other negative consequences (59). In the present study, the validation of the scale in Spanish was used (60). The internal consistency of the YFAS 2.0 in our sample was α = 0.85.

*Characteristics of eating disorders*. Eating Disorders Inventory (EDI-3) (61). The EDI-3 is a standardized measure that is easy to apply and correct, offering objective scores and profiles that are highly useful for people with characteristics or suspected diagnoses of ED. of eating behavior. It is composed of 91 items, organized into 12 main scales: 3 scales specific to EDs and 9 general psychological scales that are highly relevant to, but not specific to, ED. It also provides six indices: one specific to eating disorders (ED risk) and five integrative psychological construct indices (inefficacy, interpersonal problems, affective problems, excess control, and general psychological maladjustment). Compared to previous versions, it incorporates three validity scales that allow professionals to detect inconsistent or strange response patterns: inconsistency, infrequency, and negative impression. The total sum of the instrument measures the characteristics of the present ED. The multiple validations reflect alphas and omegas ≥0.75 for the total scale (62–64). In the present study, the instrument reported an alpha of 0.79 using the Spanish version (65).

*Disordered eating behavior*. Dutch Eating Behavior Questionnaire [DEBQ; (66)]. The English version of the DEBQ (67). The Dutch Eating Behavior Questionnaire (DEBQ) is a 33-item self-report questionnaire developed by Van Strien et al. (66) to assess three distinct eating behaviors in adults: (1) emotional eating, (2) external eating, and (3) restricted eating. Items on the DEBQ are scored from 1 (never) to 5 (very often), with higher scores indicating greater approval of the disordered eating behavior. The psychometric properties of the DEBQ are solid and show good internal consistency in non-clinical samples for the entire scale, ranging from 0.92 to 0.94 Cronbach's alpha coefficient. In the present study, an alpha of 0.89 was obtained by applying the Spanish version of the scale (68).

*Impulsivity*. The UPPS-P Impulsive Behavior Scale (69) is a 59-item scale designed to assess impulsivity, determined in five subscales: lack of deliberation, lack of perseverance, negative urgency, positive urgency, and sensation seeking. The items are evaluated from 1 (totally agree) to 4 (totally disagree). Internal consistency reliability estimates indicate that the overall scale and the subscales have an internal consistency >0.80. In the current sample, the internal consistency coefficient indicated an alpha of 0.83, respectively applied to the Spanish version (70).

*Negative affect*. The short version in Spanish of the PANAS is a self-applied instrument consisting of two sections of 20 items each, 10 of which measure positive affects and 10 negative affects. The first section evaluates the presence of affects “in recent weeks” (affect as a state), and the second section evaluates them “generally” (affect as a trait). The items are made up of words that describe different emotions and feelings and are answered by indicating a number in a range from 1 to 5, where 1 means “very little or not at all” and 5 “extremely.” The subscales of the instrument indicate good reliability, ranging from 0.75 to 0.92 for positive affect and 0.78 to 0.93 for negative affect. In the present study, an alpha of 0.78 was obtained for the negative affect and 0.80 for the positive affect, applying the Ecuadorian version (71).

*Stress*. Perceived Stress Scale [EEP; (72)]. Scale made up of 10 items that measure perceived stress—the extent to which everyday life situations are perceived as stressful. The scale includes a series of direct inquiries that explore the level of stress experienced. The paragraphs are easy to understand. The scale provides five response options: “never,” “almost never,” “occasionally,” “many times,” and “always,” which are classified from zero to four. However, items 4, 5, 7, and 8 are scored in reverse or inverted form. The EEP-4 is limited to items 2, 4, 5, and 10. The higher the score, the greater the perceived stress.

*Anxiety*. Goldberg Anxiety and Depression Scale [GADS; (73)]. This instrument is made up of two subscales of nine binary items (yes/no) each. The first anxiety subscale (questions 1–9) and the second subscale for depression (questions 10–18). Higher point values indicate greater anxiety and greater depression. The original scale and adapted versions indicate good internal consistency, ranging from 0.70 to 0.92 for both subscales. The present study sample presented Cronbach's alpha of 0.80 for the anxiety scale and 0.79 for the depression subscale, using the Ecuadorian version (74).

*Difficulties in emotion regulation:* Difficulties in Emotional Regulation Scale (DERS) (42) is a 36-item scale used to assess emotional dysregulation. The DERS consists of the following six subscales: non-acceptance of emotional responses, difficulties engaging in goal-directed behaviors when experiencing strong emotions, difficulties controlling impulses, lack of emotional awareness, limited access to emotion-regulation strategies, and lack of emotional clarity. Participants are asked to respond to each item using a five-point Likert scale ranging from 1 (almost never) to 5 (almost always). Higher scores indicate greater problems with emotion regulation. The general scale and subscales present good reliability, with alphas and omegas ranging from 0.71 to 0.86, respectively. The DERS scale has its Ecuadorian validation (75), and the total reliability of the DERS in our sample was 0.81, respectively.

### Procedure

Permission for researchers to access classrooms was granted by the Rectorate and the Teaching Department of the Catholic University of Cuenca. Researchers visited each classroom across the offered courses, introduced the study, and extended an invitation to students to participate. The study's objectives, its voluntary nature, and the data collection schedule during school hours were communicated. Only students who agreed and provided signed informed consent were evaluated on the predetermined date. During the evaluation, questionnaires were completed individually in a group setting, overseen by a seasoned psychologist. The instruments were not arranged in any specific order. To maintain privacy, participants were spaced sufficiently apart to prevent potential influence from peers' responses. Each session took about an hour. This psychologist received guidance from Spanish psychometrician colleagues. In line with the Declaration of Helsinki, the study received approval from the local Ethics Committee of the Catholic University of Cuenca under the code UCACUE-UASB-P-CEISH-2022-096 in Ecuador. All participants were required to sign informed consent.

### Statistical analysis

Statistical analysis was carried out with Stata17 for Windows. Chi-square tests (χ^2^) assessed the relationship between the categorical variables of the study (expected frequencies higher than 5 were achieved, guaranteeing the conditions for conducting the tests). The comparison between quantitative variables between the groups was done with a one-way ANOVA (which also met the assumptions of high sample sizes and common variance). It was tested that these methods fulfilled the statistical analysis assumptions in this study. The effect size for these procedures was performed with Cramer's-V coefficient for the contingency tables analyzed with χ^2^-tests and Cohen (76) *d* coefficient for the pairwise comparisons in the ANOVA.

Pearson's correlation (R) explored the relationship between the quantitative variables of the study. Due to the strong association between this model and the sample size (low-correlation values tend to achieve significant results in large samples, while high-correlation values tend to achieve non-significant results in small samples), relevant correlations were considered for |R| coefficients within the mild–moderate to large–high ranges (the cut-offs are 0.24 and 0.37, respectively) (77).

Path analysis assessed the direct and indirect links between the FA severity and the other variables of the study. In this study, this mode was implemented through the structural equation model (SEM), using the maximum-likelihood estimation (MLE) procedure, which is the most commonly used method for estimation and testing in SEMs (78). The rationale for the path diagram (i.e., the model specification) was based on the theoretical background provided by the cumulated empirical evidence (as summarized in the Introduction section), with the restrictions of the available measurement tools/data, adequate fitting and guaranteeing the clinical significance of whole model (78). All the parameters in the SEM were free estimates, and with the aim of obtaining a more parsimonious model and increasing statistical power, parameters with no significant tests were deleted, and the model was re-specified and re-adjusted (79). A goodness-of-fit was valued with the usual indexes (80): χ^2^-test, root mean squared error of approximation (RMSEA), comparative fit index (CFI), the Tucker–Lewis index (TLI), and the standardized root-mean-square residual (SRMR). Adequate fitting was considered for non-significant results in the χ^2^ test (*p* > 0.05), RMSEA <0.09, CFI > 0.90, TLI > 0.90, and SRMR <0.10. The global predictive capacity for the model was measured with the coefficient of determination (CD).

## Results

### Descriptive for the sample

Most participants in the study were women (*n* = 688, 70.8%) and single (*n* = 906, 93.2%). Mean age was 20.13 years old (SD=2.63). Table 1 displays the descriptive of the variables analyzed in the study and the comparison of the groups defined for the geographic origin. Significant differences were only observed in impulsivity and the GADS total score, with the coast group recording the highest mean values and the Galapagos group the lowest.

### Comparison between the groups defined for the FA screening

**Figure 1:**
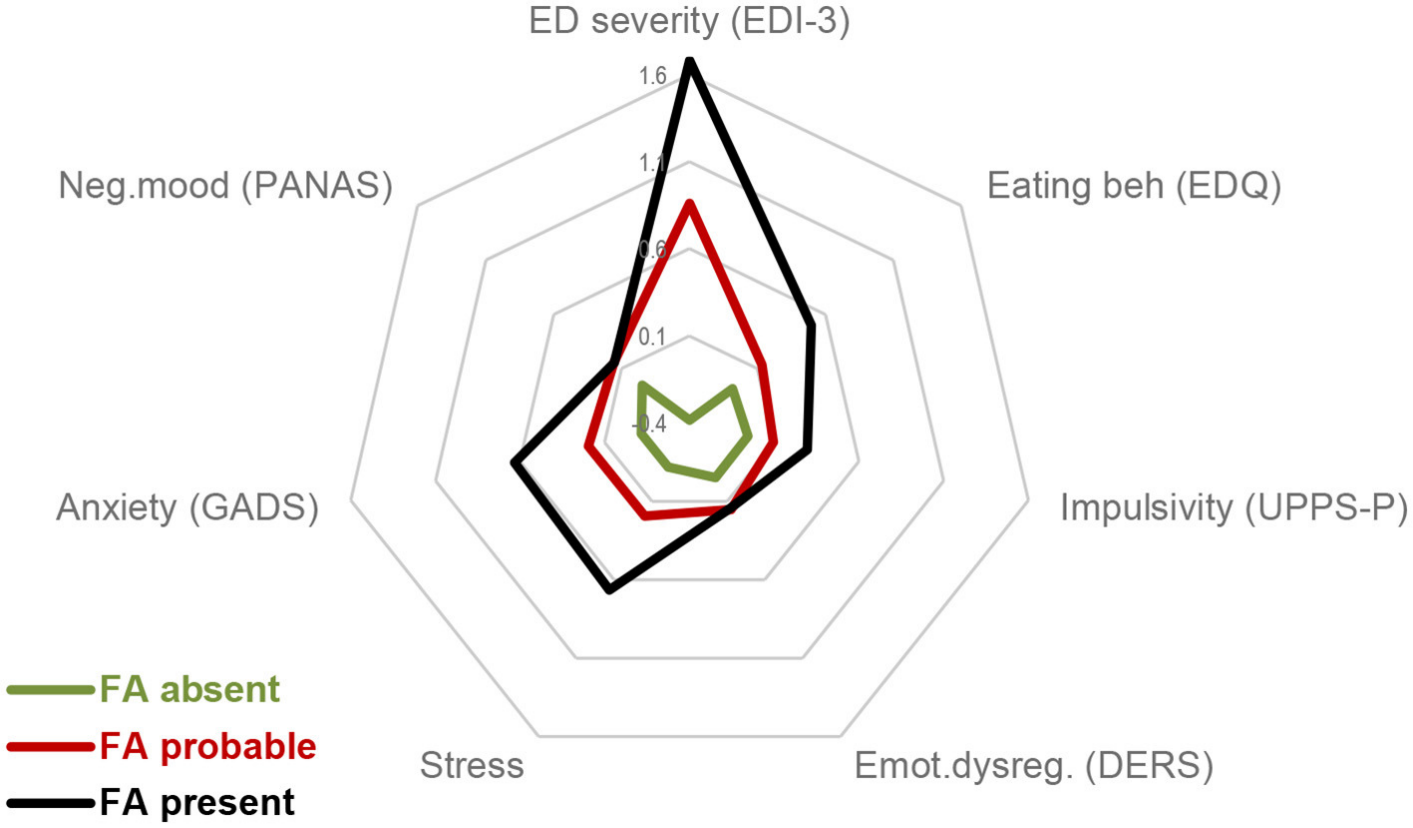
Radar chart. FA, food addiction. Standardized z-mean scores are plotted.

Table 2 shows the comparison of the participants with absent, probable, and present screening scores in the YFAS 2.0. A greater proportion contributed to the female sex, presenting high values in AF absent (71.1%), AF probable (73.3%), and AF present (62.3%) than the male sex. No differences between the groups were found for the sociodemographic features (sex, marital status, origin, and age). However, differences were obtained for the variables related to the clinical state (Figure 1). As a whole, the FA absent group was characterized by the best functional profile, with the lowest scores in the ED symptom problems (EDI-3 and DEBQ), impulsivity levels (UPPS-P), difficulties in emotion regulation (DERS), stress perceived level, anxiety (GADS), and negative emotion (PANAS). The worst functional clinical profile was related to the FA present group.

### Path analysis

**Figure 2:**
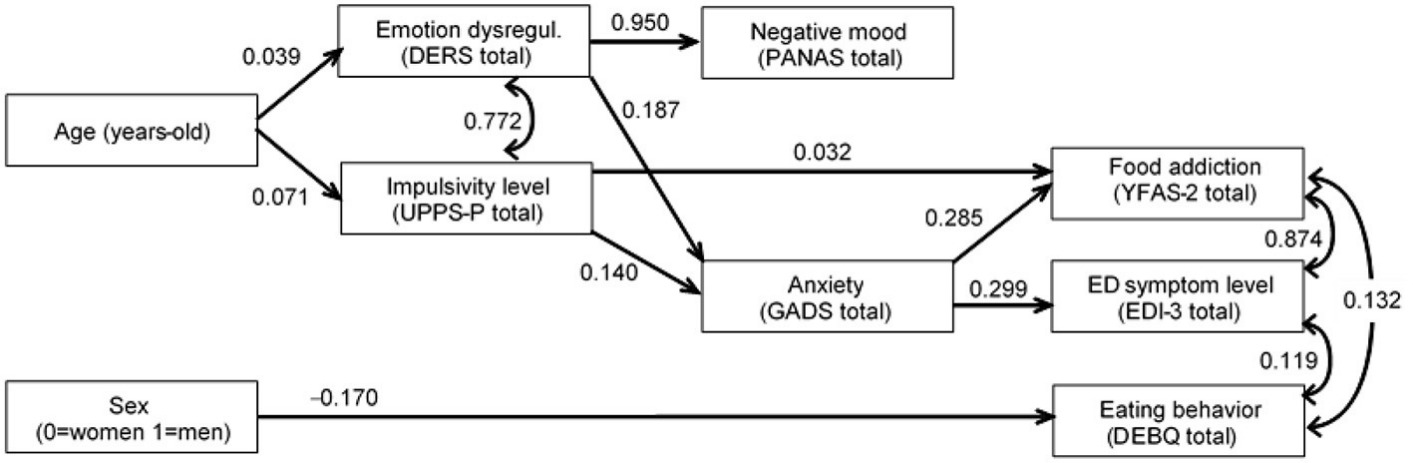
Path diagram: standardized coefficients. Only significant parameters retained in the final model. Fit statistics: χ^2^ = 16.47 (*p* = 0.792), RMSEA = 0.001 (95% CI: 0.000 to 0.018), CFI = 0.999, TLI = 0 0.998, SRMR = 0.018.

Figure 2 shows the path diagram with the standardized coefficients obtained in the SEM (Supplementary Table S1, displays the complete results, including tests for direct, indirect, and total effects). Only significant parameters were retained in the final model to allow easier interpretation. Adequate goodness-of-fit was achieved as follows: χ^2^ = 16.47 (*p* = 0.792), RMSEA = 0.001 [95% confidence interval (CI): 0.000–0.018], CFI = 0.999, TLI = 0.998, SRMR = 0.018. The global predictive capacity was CD = 0.35 (35%). No significant difference was obtained in the chi-square tests (χ^2^ = 14.96, *p* = 0.528) comparing this final model vs. the initial model that included all the direct and direct effects [χ^2^ = 1.55 (*p* = 0.959), RMSEA = 0.001, CFI = 0.999, TLI = 0.999, SRMR = 0.003, CD = 0.45].

The likelihood of higher FA levels was directly related to higher impulsivity and anxiety levels. Anxiety was also identified as a mediational link in the following two paths: (a) higher impulsivity levels contributed to a higher anxiety state and, therefore, to a more severe FA profile, and (b) higher difficulties in the emotion regulation impacted the anxiety state, and once more to a higher FA level.

Three other pathways were observed: (a) older age contributed to higher levels of impulsivity and emotion dysregulation, (b) female sex was related to more difficulties in eating behaviors, and (c) the higher the impairment in emotion regulation, the worse the negative mood state.

Finally, positive correlations were found between emotional dysregulation with impulsivity levels, FA with and ED symptoms and ED behaviors, and ED symptoms with and ED behaviors.

## Discussion

Research on FA has seen a surge in recent years, but its exploration within the Ecuadorian population remains limited. This study proposed two objectives: to assess the psychological characteristics associated with the different results of the AF screening (whether absent, probable, or present) and to formulate a predictive model that can measure the severity of FA in the Ecuadorian population.

The results of the first objective indicated that a worse psychological profile is associated with a present FA. This result is consistent with previous studies that indicate the dysfunction of the psychological areas is predominant when an eating disorder such as FA occurs (81–84). The second objective led to the formulation of a refined predictive model for the intensity of FA in the Ecuadorian populace. The findings align with existing research. Our analysis corroborates these outcomes, highlighting that FA has a positive correlation with impulsivity and anxiety. This suggests a parallel between FA and substance use disorders. Precisely, as indicated, FA and drug use disorders share analogous etiological variables such as impulsivity (85) and anxiety (30, 86). This result demonstrates that high drive levels are related to higher values in FA (87). Studies presented in this line strengthen our analysis since FA has been related to worse executive functioning and impulse control in university students, which may be a factor that contributes to a worse quality of life in this population (88–90). One aspect of impulse control that has been found to be particularly relevant to FA and eating behaviors is the tendency to have difficulty with impulse control during negative mood states (91), which was not accounted for in this study. Therefore, future research in this direction could deepen our comprehension of FA. For their part, diet and anxiety can become a recurring adverse pattern with FA (33, 92). The temptation and guilt that comes from snacking on a favorite food can lead to anxiety. While cross-sectional studies suggest an empirical connection between anxiety and FA, the direct and indirect (mediating) direction of our study's connection strengthens those results (31, 32). Studies in this line report that FA has been associated with typical anxiety and addiction phenomena such as restlessness, intense excitement, extreme insecurity, brain reward dysfunction, worry, risky consumption, impaired control, and relapse (92, 93), which would be important to address in future lines of research. Given that the literature shows that women are more prone than men to present anxiety and addiction to food and that the connection between anxiety and ED could be moderated by gender (31), Our study corroborates these findings, showing a direct influence of sex on disordered eating behavior.

In contrast, this study tested the hypotheses about the mediating role of anxiety between the indirect influence of impulsivity and emotional dysregulation on FA. Our results confirm the mediating role of anxiety in increasing ED symptoms (94), indicating that people are more likely to act impulsively when they are anxious with the certainty of increasing the probability of triggering an FA. In other words, anxiety potentiates the indirect effect of impulsivity on FA, generating a greater consumption of food. Similarly, our results confirm the role played by emotional dysregulation in EDs. Precisely, our analyses are consistent with recent studies (36, 40, 95), indicating that emotional dysregulation is significantly associated with overeating (96–98). Thus, a greater consumption of food downregulates emotional intensity, demonstrating the indirect role of emotional dysregulation on FA and symptoms associated with EDs. Moreover, dysregulation of emotions has been postulated and empirically supported as a primary transdiagnostic phenomenon across the spectrum of EDs (18, 44, 99). Finally, emotional dysregulation explains significantly more the variation in binge eating by sex, food restriction, weight assessment, and body shape (98, 100, 101). While these criteria were not explored in this study, we suggest that incorporating them in future research would enrich the analysis of the associations between emotional dysregulation and problematic eating.

Another hypothesis that was validated is the link between age, impulsivity, and emotional dysregulation. This result strengthens studies that report the role of impulsivity and emotional dysregulation during adolescence and early adulthood, precisely where there is a higher incidence of suffering from an eating disorder (22, 102–104). Furthermore, it is known that impulsivity plays an important role in addictions. Although there is situational, useful, and adaptive impulsivity, our study reflected on impulsivity as a trait frequently related to behaviors that are harmful to the individual such as inappropriate eating acts. Similar to impulsivity, several studies (105–107) corroborate our results, indicating that emotional dysregulation manages to be representative in age adolescents and young adults, essentially in the university context.

Although we did not make the direct relational hypothesis of emotion regulation and FA in our study, difficulties with emotion regulation have been shown to increase the risk of psychopathology (108–111). A review of longitudinal studies on university students investigated the relationship between emotional regulation and various EDs (112). The results suggest that dysregulation of emotions is an important factor in university students at risk of FA. Similarly, previous studies have indicated that difficulties in regulating emotions positively predicted high scores on the YFAS (22, 113) and that this strength of association is similar in patients with ED and healthy controls (43, 114). Hence, we believe these data align with the analyses presented, suggesting that behaviors associated with FA serve as a means to regulate maladaptive emotions.

According to the contrasted hypothesis on the relationship of the female sex with disordered eating behaviors, several studies ratify these results (115–117). The evidence presented to date indicates that the female sex has been very strongly associated with the presence of EDs (118). Our analysis strengthens the classic studies where the predisposition of the female sex is mentioned in the prevalence of ED, denoting it to be a predisposing factor for FA. Furthermore, we believe that this result is overshadowed by “standard factors” that are also present in EDs that were not included in this study, such as familial and socioeconomic factors (119–121).

Finally, the hypothesis on the relationship between impaired emotional regulation and negative mood was contrasted. The literature has identified both negative affect and the dysregulation of emotions as central elements of various forms of psychopathology, such as EDs (45, 47, 122, 123). Our results support these investigations and strengthen the conceptualization of emotional dysregulation as a different construct related to, but not reducible to, negative affect. Therefore, these data suggest that negative affect may be as or more strongly related to some forms of psychopathology as is the case with dysregulation of emotions in an integrated model of FA.

### Comparison with other models

Based on our findings, our model demonstrates satisfactory goodness-of-fit metrics, mirroring the model introduced by Munguía et al. (54) for FA, whose direct predictors were made up of disordered eating behavior, difficulties in emotional regulation, and impulsivity and whose indirect predictors were made up of age and the characteristics of EDs. Our study diverges by successfully incorporating negative affect, anxiety, and sex, thereby enriching the comprehension of FA within the Ecuadorian context. Similarly, our findings bolster the model presented by Wolz et al. (55), which integrates impulsivity, emotional regulation difficulties, and ED traits, resulting in a tailored SEM predictive model for FA. Other models have either examined FA independently or in tandem, weaving in predictors like impulsivity and emotional regulation (43) or age and sex (124), obtaining similarly adjusted results in the proposed models. Complementing this, a study that incorporated psychological and sociocultural variables (125) consistently indicated that the dysfunctional psychological profile emerges as a significant predictor of FA, a notion we have elaborated on extensively in our research.

### Limitations

As highlighted earlier, this marks the inaugural study in the Ecuadorian population aiming to integrate clinical variables linked to FA. Yet, it is essential to interpret these findings in light of certain limitations. Initially, the sample size was not evenly distributed across the regions examined, given that data collection occurred in its natural setting.

In addition, our study could be assessed as underpowered due to the use of path analysis. However, it must be considered that sample size requirements for SEMs rely on outdated rules of thumb and that current studies using Monte Carlo procedures that have explored requirements for some common types of models (including variation by the number of factors, the number of indicators, the strength of the indicator loadings and the regressive paths, and the amount of missing data per indicator), have evidenced that the statistical power, the bias in the parameter estimates, and the overall solution propriety, can be adequately achieved with sample sizes into a large range (from 30 to 460) (126, 127).

Moreover, the sample predominantly consisted of university students, potentially affecting the broader applicability of the findings. In a similar vein, the research focused solely on a specific age bracket, constraining the extrapolation of results to diverse age groups. Future research should thus encompass a broader spectrum of participants, spanning various age categories and additional variables to the model in question. Finally, the evaluation scales used lacked proper validation for the Ecuadorian demographic.

## Conclusion

We deduce that elevated psychopathological levels correlate with food addiction. Additionally, there is no discernible difference in the risk of FA across Ecuadorian regions. In conclusion, our analysis indicates that older age, being female, and exhibiting heightened levels of emotional dysregulation, impulsivity, negative affect, and anxiety can precipitate EDs and, as a result, FA in the Ecuadorian population.

## Data availability statement

The original contributions presented in the study are included in the article/Supplementary material, further inquiries can be directed to the corresponding author.

## Ethics statement

The studies involving humans were approved by the Ethics Committee of the Catholic University of Cuenca with the code UCACUE-UASB-P-CEISH-2022-096 - in Ecuador. The studies were conducted in accordance with the local legislation and institutional requirements. Written informed consent for participation in this study was provided by the participants' legal guardians/next of kin.

## Author contributions

GR did the original writing and drafting. RG developed the methodology, data analysis, and results. XC, JV, JE, and RY recruited and processed the data. All authors contributed to the conceptualization, supervision, writing, revision, and approval of the final version of the manuscript.
