# Supplementary material for: Factors increasing the risk for food addiction in Ecuadorian students
Source: Front Psychiatry. 2024 Jan 3;14:1214266. doi: 10.3389/fpsyt.2023.1214266 (PMC10792013; doi:10.3389/fpsyt.2023.1214266)
Supplement: Supplementary file 1 [file Table_1.pdf]

***Table S1 (supplementary)*** Test for direct, indirect and total effects in the SEM

| *Direct effects* | | *B* | *SE* | *z-statistic* | *p* | *Stand.B* |
|---|---|---|---|---|---|---|
| DEBQ total | Sex | -0.5149 | 0.0949 | -5.42 | <.001 | -.1701 |
| UPPS-P total | Age | 0.2210 | 0.1003 | 2.20 | .028 | .0705 |
| DERS total | Age | 0.0942 | 0.0777 | 1.21 | .225 | .0389 |
| GADS total | UPPS total | 0.0838 | 0.0287 | 2.92 | .004 | .1402 |
| | DERS total | 0.1448 | 0.0372 | 3.90 | <.001 | .1872 |
| YFAS total | UPPS-P total | 0.0728 | 0.0349 | 2.09 | .037 | .0323 |
| | GADS total | 1.0798 | 0.1156 | 9.34 | <.001 | .2867 |
| PANAS total | DERS total | 0.4847 | 0.0051 | 94.75 | <.001 | .9499 |
| EDI-3 total | GADS total | 2.2638 | 0.2362 | 9.58 | <.001 | .2920 |
| *Indirect effects* | | *B* | *SE* | *z-statistic* | *p* | *Stand.B* |
| GADS total | Age | 0.0322 | 0.0190 | 1.69 | .091 | .0172 |
| YFAS total | UPPS-P total | 0.0905 | 0.0325 | 2.79 | .005 | .0402 |
| | DERS total | 0.1563 | 0.0435 | 3.60 | <.001 | .0537 |
| | Age | 0.0508 | 0.0285 | 1.79 | .074 | .0072 |
| PANAS total | Age | 0.0457 | 0.0377 | 1.21 | .225 | .0369 |
| EDI--3 total | UPPS total | 0.1898 | 0.0680 | 2.79 | .005 | .0409 |
| | DERS total | 0.3277 | 0.0908 | 3.61 | <.001 | .0547 |
| | Age | 0.0728 | 0.0437 | 1.67 | .096 | .0050 |
| *Total effects* | | *B* | *SE* | *z-statistic* | *p* | *Stand.B* |
| DEBQ total | Sex | -0.5149 | 0.0949 | -5.42 | <.001 | -.1701 |
| UPPS total | Age | 0.2210 | 0.1003 | 2.20 | .028 | .0705 |
| DERS total | Age | 0.0942 | 0.0777 | 1.21 | .225 | .0389 |
| GADS total | UPPS-P total | 0.0838 | 0.0287 | 2.92 | .004 | .1402 |
| | DERS total | 0.1448 | 0.0372 | 3.90 | <.001 | .1872 |
| | Age | 0.0322 | 0.0190 | 1.69 | .091 | .0172 |
| YFAS total | UPPS-P total | 0.1634 | 0.0467 | 3.50 | <.001 | .0725 |
| | DERS total | 0.1563 | 0.0435 | 3.60 | <.001 | .0537 |
| | GADS total | 1.0798 | 0.1156 | 9.34 | <.001 | .2867 |
| | Age | 0.0508 | 0.0285 | 1.79 | .074 | .0072 |
| PANAS total | DERS total | 0.4847 | 0.0051 | 94.75 | <.001 | .9499 |
| | Age | 0.0457 | 0.0377 | 1.21 | .225 | .0369 |
| EDI-3 total | UPPS-P total | 0.1898 | 0.0680 | 2.79 | .005 | .0409 |
| | DERS total | 0.3277 | 0.0908 | 3.61 | <.001 | .0547 |
| | GADS total | 2.2638 | 0.2362 | 9.58 | <.001 | .2920 |
| | Age | 0.0728 | 0.0437 | 1.67 | .096 | .0050 |

*Note*. B: non-standardized coefficient. SE: standard error. Stand.B: standardized coefficient. EDI: Eating Disorders Inventory; DEBQ: Dutch Eating Behavior Questionnaire; UPPS-P: Impulsive Behavior Scale; DERS: Difficulties in Emotional Regulation Scale; PSS: Perceived Stress Scale; GADS: Goldberg Anxiety and Depression Scale; PANAS: Positive and Negative Affect Scale; YFAS: Yale Food Addiction Scale.
